# CBCT Assessment for Dental Implant Surgery at the Maxilla: A Clinical Update

**DOI:** 10.3390/diagnostics16030479

**Published:** 2026-02-04

**Authors:** Wai Yu Chelsea Chung, Feng Wang, Yiu Yan Leung

**Affiliations:** Oral & Maxillofacial Surgery, Faculty of Dentistry, The University of Hong Kong, Hong Kong SAR, China; chel368@connect.hku.hk (W.Y.C.C.); diawang@hku.hk (F.W.)

**Keywords:** dental implant, cone-beam computed tomography (CBCT), maxilla, risk assessment, bone augmentation

## Abstract

In contemporary practice, dental implants are widely recognized as a reliable and effective solution for rehabilitating edentulous patients. Nevertheless, implant placement in the atrophied maxilla presents considerable challenges, with treatment planning influenced by various factors such as patient demographics, anatomical constraints, and economic considerations. Advances in imaging technology have positioned cone-beam computed tomography (CBCT) as the preferred modality for enhancing implant placement accuracy. By producing high-resolution three-dimensional radiographic images, CBCT facilitates precise assessment of maxillary anatomy at the proposed implant site—including bone height, width, length, and angulation—thereby optimizing surgical planning and improving the predictability and success rates of implant integration. Moreover, the timing of implant placement must account for the necessity of maxillary augmentation to ensure implant stability and reduce the risk of postoperative complications. This review discusses the clinical utility of CBCT as a diagnostic tool for preoperative assessment, focusing on the identification of critical anatomical landmarks and the determination of indications for bone augmentation, thereby highlighting its crucial role in enabling accurate treatment planning, minimizing surgical risks, and promoting the long-term survival of dental implants.

## 1. Introduction

As dental implants have become an increasingly popular treatment choice for edentulism, bone augmentation is an indispensable technique for implant placement in the atrophic maxilla. Since Brånemark’s unintentional discovery of osseointegration was applied to the development of dental implants in 1952, prosthetic dental rehabilitation has a complete paradigm shift for a definitive, enduring, and clinically robust replacement of missing dentition [1].

Comprehensive clinical and radiographic evaluations are essential prior to dental implant placement to assess systemic and local suitability.

Systemic factors such as the use of bisphosphonates or other antiresorptive drugs for osteoporosis, as well as immunotherapy and radiotherapy for head and neck cancer, represent relative contraindications for implant therapy and may reduce implant survival. Additional conditions, including uncontrolled diabetes, Crohn’s disease, and smoking, increase the risk of peri-implantitis, further compromising implant longevity [2,3].

Locally, inadequate bone volume or quality is linked to impaired osseointegration and implant failure [4], while smoking and excessive alcohol consumption are correlated with an increased prevalence of peri-implantitis [5,6]. Treatment outcomes are also influenced by local factors including implant site selection, surgical technique, and prosthetic planning [7], with improper implant positioning [8], compromised alveolar bone quality [9], and prior site-specific surgical interventions such as bone grafting or sinus augmentation [10] identified as risk factors for postoperative complications [11]. Accordingly, three-dimensional radiographic assessment is recommended to facilitate accurate diagnosis and individualized treatment planning.

Cone-beam computed tomography (CBCT) has become the imaging modality of choice for dental implant treatment due to its ability to generate high-resolution volumetric images of the teeth and alveolar bone at a justifiable cost and relatively low radiation dose, owing to its limited field of view. Preoperative assessment of the alveolar bone is critical for predicting implant success [12], particularly in the maxilla, where the proximity of planned implant sites to vital anatomical structures must be carefully evaluated. CBCT enables accurate analysis of alveolar bone quantity, including height and thickness, and bone quality, assessed by density in Hounsfield units and classified according to cortical thickness and trabecular bone characteristics under the Lekholm and Zarb system [13]. Radiographic evaluation using CBCT, in conjunction with panoramic imaging, also guides the decision to incorporate bone augmentation into treatment planning [14], with horizontal augmentation indicated for buccolingual or mesiodistal ridge deficiencies [15] and vertical augmentation recommended for apicocoronal bone loss to restore adequate alveolar height and facilitate optimal implant positioning [16]. Accordingly, thorough clinical diagnosis and meticulous treatment planning, integrating both systemic and local factors, are essential to promote successful osseointegration and favorable implant outcomes.

With dental implants representing the mainstream choice of tooth replacement in contemporary dentistry, it is essential to conduct comprehensive preoperative assessments and evaluate patient-specific anatomical considerations to facilitate optimal implant placement and long-term clinical success. In this review, we discuss the importance of the use of CBCT as an assessment tool for dental implant surgery, followed by clinical updates in dental implant placement in the maxilla.

## 2. Radiographic Features of the Maxilla on CBCT

In light of substantial clinical evidence, anatomical features acquired from CBCT images that are pertinent to dental implant placement, could, in fact, remain undetected with panoramic radiography [17]. To minimize surgical complications and implant placement errors, CBCT has become a routine diagnostic tool for dental implant surgery [18].

### 2.1. Nasopalatine Canal

Not only are the location and angulation of implant placement important, but the anchorage which determines the primary stability of the implant placed, also serves as a decisive factor in determining the overall success of the surgery [19]. The nasopalatine canal which houses the nasopalatine neurovascular bundle, is a corticated structure [20]. In patients with a heavily atrophied maxillary crestal ridge, the placement of dental implant within the cortical bone of the nasopalatine canal area for primary anchorage establishment is advocated to ensure long-term success, thus accurate measurement of the canal dimensions and the careful selection of implant type are crucial. However, misplacement of dental implant in the nasopalatine canal could result in complications like bleeding, sensory disturbance, and lack of osseointegration, which could potentially culminate in surgical failure [19].

### 2.2. Canalis Sinuosus

Branched from the infraorbital canal, the canalis sinuosus is a bony canal that houses the anterior superior alveolar nerve and vessels supplying the maxillary anteriors [21]. The difficulty in identifying the canalis sinuosus often comes with clinicians’ inexperience and radiographic scans with low resolution [22]. Given a prevalence of 36.9%, particularly in the Chinese population, the orientation and depth of implants placed in the maxillary alveolar bone should be precisely assessed on CBCT images, and surgical procedures should be performed with heightened vigilance to prevent neurovascular injuries [23].

### 2.3. Maxillary Sinus

A thorough understanding of the vascularization network of the maxillary sinus, especially the arterial diameter and its proximity to the alveolar crest, aids in evaluating the risks of excessive bleeding and prevention of complications during dental implant surgery. The maxillary sinus mucosa is supplied by an extensive vascular network with three primary arterial contributions, the posterior superior alveolar artery (PSAA), the infraorbital artery (IOA), and the posterior lateral nasal artery (PLNA) [24,25]. With the PSAA running along the lateral wall of the maxillary sinus, it typically appears as a hypodense canal on the CBCT image [26], where it could be visualized most clearly in the coronal section [27]. Together with the IOA, they form an intricate network, most commonly intraosseous branches, that connect and supply the Schneiderian membrane and the lateral wall of the maxillary sinus [28]. The PLNA comprises the major blood supply to the inferior turbinate, which provides a significant amount of soft tissue available for use in regional flap reconstruction involved in sinus augmentation [29].

Sensory innervation of the maxillary sinus is provided by terminal branches of the maxillary division (V2) of the trigeminal nerve [30]. While neurosensory disturbances and incidences of nerve injury are inconsistent and less likely to occur in the maxillary implant placement, patients could experience symptoms with differing magnitudes: numbness to cheek, and lips, impaired verbal expression, masticatory problems, uncontrollable facial muscles leading to unintended drooling, and infrequently, chronic pain [31].

**Figure 1:**
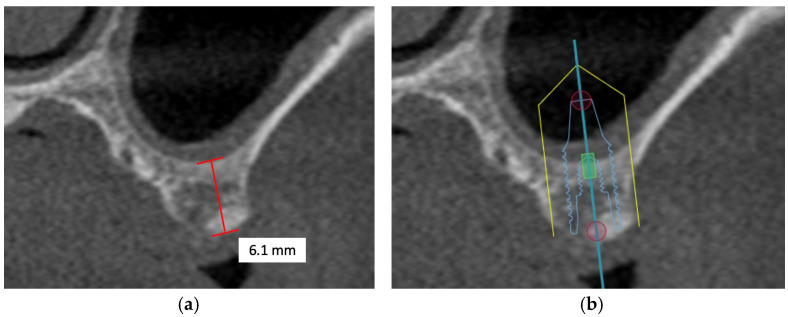
The CBCT image illustrates a pneumatized maxillary sinus in the context of implant planning: (**a**) evidence of sinus pneumatization is visible; (**b**) the proposed implant trajectory intrudes upon the expanded maxillary sinus, so maxillary sinus augmentation is necessary.

Common maxillary sinus anatomic variants include pneumatization, antral septa, hypoplasia and accessory maxillary ostium, and mucosal thickening [32]. It is critically important to identify and inspect all structural anomalies during examination and treatment planning to prevent complications, such as sinus membrane perforations and profuse bleeding. Maxillary sinus pneumatization is the most common anatomical variation as found across the literature, suggesting that tooth loss without timely replacement contributes to an elevated post-physiological risk of pneumatization [33] (Figure 1). Provoked by posterior tooth extraction, alveolar ridge remodeling leads to an increase in the maxillary sinus size [34]. Thus, it adds complexity to dental implant surgery, as internal or external sinus elevation may be necessary in atrophic maxillae—a factor that could be easily overlooked in the absence of CBCT [35]. As a result of pneumatization, antral septa—thin, fragile, and sickle-shaped walls, can develop within the maxillary sinus, resulting in compartmentalization. Coupled with total compartmentalization of the sinus, it can give rise to a 8.6% accidental perforation of the Schneiderian membrane during sinus floor elevation, in which surgeons would need to resort to alterations of the lateral sinus elevation [36]. Maxillary sinus hypoplasia (MSH), primarily caused by infections, irradiation, or syndromes, is commonly detected on the ipsilateral side corresponding to the patient’s potentially deviated nasal septum [37,38]. Typically, MSH does not complicate dental implant surgeries; notwithstanding, certain variants like inferior meatus pneumatization (characterized by an enlarged inferior meatus, the boundary between the maxillary sinus and the nasal cavity is displaced laterally from its normal anatomical position) and ethmomaxillary sinus (a rare anatomical variant characterized by the extension of the posterior ethmoid air cells into the maxillary sinus), could pose challenges to surgical planning and the management of complications [39]. Accessory maxillary ostium (AMO), located between the uncinate process and the inferior concha, is a maxillary sinus perforations in the fontanelles caused from maxillary sinusitis. For successful dental implant placement and postoperative management, sinus membrane elevation is necessary to ensure sufficient amount of alveolar bone can be grafted, resulting in uneventful osseointegration of the implant with regenerated bone. To preserve the physiological functions of the maxillary sinus following dental implant surgery, rigorous CBCT analysis of the location and dimension of the AMO should be done preoperatively [40]. Mucosal thickening in the maxillary sinus, often appearing as a hazy or opaque line on radiographs, is possibly caused by odontogenic irritations like trauma to the maxillofacial region, infections of the nasal conchae, and hypersensitivity to non-infectious particles, and could drastically compromise the prognosis of the placed dental implant [41].

Moreover, maxillary sinus pathologies, such as maxillary sinusitis, may complicate dental implant surgery due to implant malpositioning and have a reported prevalence of 18.3%, potentially necessitating additional treatments before implant placement [42,43]. With the maxillary sinus in correlation with the ethmoid sinus because they share the osteomeatal unit, sinus pathologies do not present any association with anatomical variations in the ethmoid sinuses [44]. Assessment of the maxillary sinus should be made for detection of both anatomical variations as mentioned, as well as any sign of pathological changes that may lead to a compromised outcome after implant placement.

In sum, radiographic images produced by CBCT of high diagnostic quality allow precise measurements to be made from the alveolar ridge to the maxillary sinus floor to calculate the maximum depth of dental implant that could be placed at the edentulous site.

### 2.4. Antral Alveolar Artery

The antral alveolar artery (AAA), located between the posterior superior alveolar artery (PSAA) and the infraorbital artery (IOA), should be carefully identified during lateral sinus lift procedures, as its presence may interfere with the surgical window in approximately 20% of cases and increase the risk of intraoperative bleeding [45]. AAA provides blood supply to the maxillary sinus mucous membrane and the maxillary periosteum. Progressive enlargement of the artery elevates the likelihood of hemorrhage, thereby creating the potential need for litigation and jeopardy of surgical outcomes [45]. Volumetric CBCT images allow the evaluation of both the diameter and the course of the AAA [46], enabling surgeons to undertake comprehensive preoperative planning prior to dental implant placement.

### 2.5. Concha Bullosa

With a high prevalence of 30.4%, the most frequently observed incidental finding (IF) is the concha bullosa. As it could easily be overlooked from low resolution radiographs, CBCT with a suitable FOV should be used for accurate identification of IFs [31]. The concha bullosa acts as a potential contributor to the development of inferior pneumatization of the maxillary sinus. With the aid of CBCT, the investigation of the intricate anatomy of the maxillary sinuses could be made possible, enabling clinicians to anticipate and avert the possible risk of rhinosinusitis associated with the narrowed osteomeatal complex [47].

## 3. Pretreatment Assessment by Radiographic Imaging

To achieve comprehensive pre-surgical assessment, both clinical and radiographic examinations constitute significant factors in proper treatment planning. While the condition of the alveolar bone and the surrounding structures cannot be adequately assessed clinically, radiographic images with clearly captured anatomical features should be produced for accurate diagnosis.

Since the emergence of dental implants, various imaging modalities have been employed as essential tools for preoperative assessment, with panoramic radiography and CBCT being the most common modalities of choice [48]. Panoramic radiography (PR) is highly regarded as the most requested imaging exam by dental surgeons due to its ease of access, lower radiation dose and cost, and for enabling the visualization of neighboring teeth and supporting structures in a single image. Yet, PR inaccuracies have been documented to include variable vertical and horizontal magnification factors, projection geometry, focal trough depth and geometry, and positioning errors of the patient [49]. Although some studies have reported that PR was considered efficient when compared to CBCT without statistically significant difference in the treatment decision to be made [50], but it is still an unreliable method to determine the relationship between the implant site and its surrounding vital structures owing to angular distortion [51]. Watanabe et al. (2020) [52] reported that an irregular alveolar recess of the maxillary sinus may be erroneously interpreted as a sinus lesion when assessed using two-dimensional (2D) image-based algorithms, while extensive lesions in the posterior maxilla can be misclassified as part of the maxillary sinus owing to the superimposition of these structures in 2D imaging [53]. Nonetheless, in patients in whom bone quality and implant site can be accurately assessed clinically, CBCT may not be necessary as a supplemental pre-treatment radiograph. The choice of imaging modality should instead be guided by the clinician’s tactile assessment and clinical experience in treatment planning [54].

CBCT is the recommended tool for preoperative evaluation of compromised implant sites with severe alveolar resorption, allowing accurate assessment and classification of both bone quality and implant site during surgical planning. As compared with PR, CBCT is able to provide images with high spatial resolution at a relatively low radiation dose and minimal image magnification that could increase diagnostic accuracy and thus, appropriate treatment selection could be accomplished. In cases where bone augmentation is strongly indicated, clear three-dimensional visualization of the alveolar bone is critical for accurate treatment planning. Nonetheless, the decision to perform axial views, defined as horizontal sections encompassing the implant model platform, and cross-sectional views, defined as sections perpendicular to the tangent of the dental arch and passing through the center of the implant model [55], in three-dimensional imaging should be guided by clearly defined clinical and surgical requirements, in accordance with the ALARA (as low as reasonably achievable) principle [56].

To balance the patient’s radiation exposure and the diagnostic need, protocols for maxillary implant planning need to be diligently adjusted to ensure that the benefits outweigh the risks.

With the aim of balancing the diagnostic quality of CBCT imaging with radiation dose, a proof-of-concept study conducted by Tadinada (2023) [57] demonstrated that a lower-dose, modified-arc 180° CBCT acquisition protocol, approximately 55% of the full rotation, produced pre-treatment diagnostic outcomes comparable to those of the conventional 360° protocol. A drastic reduction in radiation exposure associated with the 180° CBCT protocol is achieved through shortened exposure time and optimized X-ray tube positioning, which together provide additional protection to surrounding organs [57]. A small field of view (FOV) CBCT scan, representing sextants with cylindrical dimensions of 50 mm in diameter and 37.5 mm in height, has demonstrated minimal deviation in planned anterior implant positioning in patients with sufficient, well-distributed dentition. However, due to increased angular and linear deviations in the buccolingual direction in the posterior maxilla, the use of a larger FOV has been suggested, as the greater curvature of the dental arch captured in the anterior FOV provides improved tripodization [58]. CBCT scouting also plays an important role in ensuring accurate patient positioning prior to exposing the patient to a higher radiation dose during the full diagnostic CBCT acquisition. Voxel size directly influences spatial resolution and plays a key role in balancing image detail with radiation exposure. In implantology, where linear measurements are generally sufficient, voxel size should not be overemphasized as a determining parameter prior to CBCT acquisition. When appropriate, the use of larger voxel sizes (e.g., 0.40 mm) may be recommended, as they allow shorter reconstruction times and smaller file sizes and may, in some cases, reduce radiation dose without compromising the quality of clinical treatment planning [59]. Radiographic exposure parameters play an important role in determining CBCT image quality and, consequently, measurement accuracy. Higher tube voltage (kV) is associated with lower mean squared measurement error, while measurement distortion is influenced by both bone margin thickness and the distance over which measurements are taken, with greater thickness and longer distances resulting in increased error. Accurate assessment of bone margin thickness can be achieved within a tube voltage range of 70–90 kV. In contrast, variations in tube current (mA) have not been shown to significantly influence image quality or measurement accuracy and therefore primarily affect radiation dose rather than diagnostic performance [60].

In the present era, digital implant dentistry enables the integration of cutting-edge technologies to optimize the planning and surgical placement of dental implants when clinical assessment and PR alone is inadequate. As an extended application of CBCT, “registration” aids the clinician in comparing the planned implant position preoperatively with the inserted definitive restoration postoperatively. Three-dimensional data, in particular on the quantitative composition of the gingiva, can be superimposed in digital form with CBCT data to create a virtual model of hard and soft tissue, which plays an important role in implant surgery and implant-prosthetic therapy [61]. This approach facilitates preoperative surgical planning by providing clinicians with precise visualization of the predicted postoperative outcome prior to the procedure. By superimposing the postoperative radiograph on the preoperative image with a correct spatial relationship between the two images, the anterior facial bone morphology of the multilayered virtual patient could be evaluated, further enhancing accuracy of depth and angulation of implant placement. Optimal registration of datasets permits simultaneous visualization of anatomical structures and of the planned surgical or prosthetic information, while reducing the accumulation of errors in the digital workflow that could lead to an increased discrepancy between the planned and the actual implant positions [62]. Digital diagnostic imaging software enables advanced image reconstruction and accurate measurement of CBCT data [63], thereby improving surgical precision and reducing the risk of postoperative complications. As digital dentistry has become increasingly integral to contemporary treatment planning, such software offers distinct benefits across varying levels of clinical experience. Experienced surgeons may rely on digital imaging tools primarily in complex cases where enhanced visualization of anatomical structures can substantially improve surgical accuracy, whereas less experienced surgeons may benefit from more frequent use of CBCT-based digital planning to support anatomical interpretation and decision-making during their early clinical practice. Accordingly, the selective and experience-dependent use of digital CBCT imaging software plays a critical role in optimizing pre-treatment assessment and supporting accurate, safe, and predictable implant treatment planning.

## 4. Indications of Maxillary Bone Augmentation as Determined from CBCT

While immediate implant placement in post-extraction sites in a single-session surgery guarantees enhanced aesthetic results with high success rates, ranging from 92.7% to 98% [64], a high percentage (up to 94.1%) [65] of implant placements is delayed for various reasons, such as patient demographics, anatomical considerations, and economic factors.

Studies have provided evidence that the amount of keratinized mucosa width (KMW), which denotes the height of keratinized soft tissue that runs apico-coronally from the mucosal margin to the mucogingival junction, determines the amount of soft-tissue recession and extent of bone resorption after implant placement. Three-dimensional imaging modalities for the evaluation, quantification, and diagnosis of peri-implant soft tissues prior to implant placement are predominantly based on CBCT. The subsequent integration of CBCT datasets with optical surface scans through digital superimposition permits highly precise three-dimensional characterization of soft tissue thickness [66]. With “adequate” KMW defined as ≥2 mm, patients with KMW < 2 mm are unable to perform oral hygiene practices that are up to par, reflected from a significantly higher plaque score. A plausible explanation for these findings is that a shallow vestibule may impede adequate access in the absence of KMW, while the lack of keratinized mucosa may further contribute to increased discomfort during toothbrushing. Although a 2 mm threshold has been the most employed cutoff in the current literature, the minimum width of KMW required to sustain optimal peri-implant health likely depends on various site-specific characteristics of each individual case, including mucosal thickness, supracrestal tissue height, and peri-implant bone thickness [67]. As postoperative maintenance of alveolar bone and soft tissue health around implants is important in sustaining its longevity, various surgical procedures must be carried out at sites with insufficient KMW to preserve and/or reconstruct keratinized tissue around dental implants, including apically positioned flaps, pedicle graft, free gingival graft, and connective tissue graft [68].

One of the most notable aspects—the amount of alveolar bone loss—is commonly linked with traumatic tooth extraction, trauma, pathologies, or periodontitis [69]. Studies have shown that the unavoidable resorption of bundle bone and external bone remodeling following tooth loss leads to a reduction in bone dimension of the alveolar ridge due to the increased osteoclastic activity [70]. The lack of blood supply may contribute to significant bone loss, especially in the thin buccal bone plate, 6 months after tooth extraction [71]. Thus, replacement and regeneration of lost bone prior to implant placement require grafting.

The assessment of bone density values derived from CBCT images enables modification of both surgical protocols, including drilling sequence, insertion torque, implant size selection, and the number of implants placed, and prosthetic protocols, such as healing duration and progressive bone loading. Several aspects need to be considered while planning implant placement using CBCT, including the angulation and thickness of the surrounding alveolar bone, the proximity of nearby anatomical structures, and the bone architecture around the implant, which might have implications for the long-term success of the implant [56].

Recent advancements in CBCT technology have markedly improved the accuracy and efficiency of surgical planning, particularly in complex cases requiring detailed three-dimensional assessment of maxillary anatomy. Although CBCT enables precise evaluation of alveolar bone dimensions and supports predictable implant and augmentation outcomes, its routine use is not mandatory in all clinical scenarios. In straightforward cases managed by experienced clinicians, conventional clinical and radiographic assessment may be sufficient, even when bone augmentation is anticipated. Nevertheless, in the presence of increased anatomical complexity, diagnostic uncertainty, or when a more conservative approach is desired, CBCT is recommended to enhance diagnostic confidence and ensure patient safety.

### 4.1. Available Bone Height

The remaining alveolar bone height is the most significant criterion affecting long-term survival of endosteal implants as it affects both the implant length and crown height. Measured from the crest of the edentulous ridge to the opposing landmark, the anterior regions are limited by the maxillary nerve, and the posterior regions are limited by the maxillary sinus. The canine eminence provides the greatest anterior jaw height, while the first premolar region provides the greatest posterior jaw height [63]. With sufficient alveolar bone height, placement of sub-crestal implants results in effective attenuation of biological complications of crestal bone loss, with implant–abutment interface isolated from oral pathogens, and a reduction in the risk of inflammation or infection follows [72,73].

**Figure 2:**
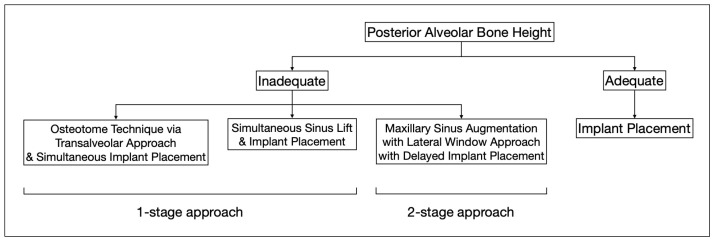
This flowchart depicts the distinct treatment strategies for cases with adequate and inadequate posterior alveolar bone height.

However, in patients with severe bone defects, rehabilitation in the maxillary anterior teeth—the aesthetic zone, is particularly challenging. With the currently available options in modern dentistry, the shortest implant of 6 mm can be placed to avoid the requirements for bone grafting [74]. As proposed by Mistry, A. et al. (2021) [75] and Renton et al. (2010) [76], if a safety zone of at least 2 mm between the planned position of the implant apex and the vital structures is not available, bone augmentation with autogenous bone grafts followed by implant placement is the gold standard treatment for such patients in order to maximize the implant longevity and the final aesthetic outcome [77]. Posteriorly, sinus augmentation is indicated when a patient presents with an edentulous and atrophic maxillary alveolus that cannot provide adequate support to the implant [31] (Figure 2).

### 4.2. Available Bone Width

The width of the alveolar bone is measured between the buccal and palatal plates at the crest of the potential implant site. Morphological differences between the anterior and posterior maxilla should be considered. The crest of the posterior maxillary edentulous ridge is typically supported by a wider base, unlike the anterior maxilla, where a labial concavity is observed in the incisor area, demonstrating an hourglass configuration. Thus, the true diameter of the implant at the crest module should always be determined after CBCT evaluation of the ridge morphology to avoid placement errors and consequential complications [63].

**Figure 3:**
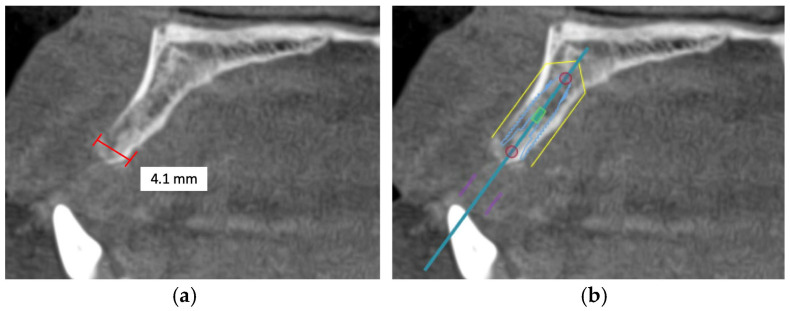
This CBCT image demonstrates a limited maxillary alveolar bone width pertinent to implant planning: (**a**) the alveolar ridge exhibits reduced width, which may be inadequate for the demands of implant placement; (**b**) given that the intended implant position exceeds the alveolar ridge width, horizontal bone augmentation is necessary.

Alveolar bone width should exceed the diameter of the proposed implant by 1.0 to 1.5 mm on the buccal and lingual or palatal side. Ridge width should be 6.0 to 7.0 mm for standard diameter implants, 5.0 to 6.0 mm for reduced diameter implants, and at least 7.5 mm for wide implants [78]. Under circumstances in which there is inadequate maxillary alveolar ridge width, horizontal bone augmentation, such as onlay grafting, ridge splitting, and guided bone regeneration using resorbable or non-resorbable barriers, is indicated to achieve desirable bone parameters prior to implant placement [79] (Figure 3).

### 4.3. Available Bone Length

The mesiodistal length of available bone in an edentulous area is often limited by adjacent teeth or implants [63]. To minimize the risk of damage to the adjacent teeth, such as tooth intrusion and interproximal contact loss [78], a distance of at least 1.5 mm must be maintained from the implant. Additionally, as proposed by Andre, A., & Ogle, O. E. (2021), soft tissue health should also be considered, with a recommended distance of 2.0 mm between the implant neck and the crown of the adjacent tooth to reduce biological complications [80]. A minimum distance of 3.0 mm between adjacent implants is advised to lower the risk of peri-implantitis and to guide appropriate implant diameter selection. Given that the smallest implant diameter is 1.8 mm [81], the mesiodistal length of the edentulous space should be at least 4.8 mm to allow for implant placement.

In single-tooth replacements, the varying lengths of the edentulous span also determine the number of implants that may be inserted. Optimization of stress distribution at the bone-implant interface is critical to enhancing the long-term biomechanical performance of the dental implant system [82]. For short edentulous spans, insertion of a single implant with a standard diameter is preferred over placing two implants due to the proximity of the site of force application [83]. Conversely, for optimal stress distribution and minimization of peri-implant crestal bone loss, increasing the number of dental implants to three reduces the stress applied to each implant and the surrounding bone in long edentulous spans [84]. In cases of insufficient ridge length for implant placement, horizontal alveolar ridge splitting or expansion allows the atrophied maxilla to be augmented and space created for implant embedment [85].

In addition, aesthetic considerations must be a high priority when implants are placed in the anterior maxilla. Ideally, the implant diameter should correspond to the natural tooth width measured 2.0 apical to the CEJ to mimic a natural implant crown emergence profile, while maintaining a minimum distance of 1.5 mm from adjacent teeth or 3.0 mm from adjacent implants [63].

### 4.4. Available Bone Angulation

The initial alveolar bone angulation represents the natural tooth root trajectory in relation to the occlusal plane. Implants should be placed perpendicularly to the curve of Wilson and the curve of Spee [63]. The larger the angle at which the implant is inserted, the higher the likelihood of resultant progressive peri-implant bone loss, with non-axially positioned implants exhibiting greater bone loss compared with axially positioned implants [86]. While crestal stress increases with implant angulation, anatomic limitations may require intentional tilting.

In patients with a narrow maxillary alveolar ridge, mispositioning of the implant may easily result from miscalculation of the alveolar bone dimensions and technical errors in projecting of the implant in the maxilla during pre-operative surgical planning. Advantageously, the repositioning of dental implants has been made possible with segmental osteotomy after osseointegration, allowing for an anatomically and physiologically favorable occlusal function [87].

## 5. CBCT-Directed Approaches to Maxillary Bone Regeneration

In clinical practice, CBCT is frequently used to assess the dimensions and health of the alveolar ridge and maxillary sinus, supporting implant planning, sinus lift procedures, and the determination of required bone graft volume [88]. The variable quality and quantity of maxillary bone evident on CBCT images can pose challenges for implant placement, but CBCT remains one of the most effective modalities for evaluating residual bone morphology and quality, providing valuable information on the distribution of cortical and cancellous bone in the maxilla.

**Figure 4:**
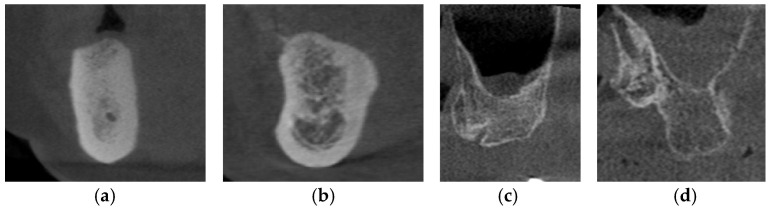
The CBCT images depict the various alveolar bone classifications: (**a**) Type I; (**b**) Type II; (**c**) Type III; and (**d**) Type IV.

The Lekholm and Zarb classification continues to be the most frequently cited and extensively applied system for bone quality evaluation in the literature [89]. According to the classification as proposed, the quality of residual alveolar bone is categorized into four distinct types: Type I—characterized by predominantly homogeneous cortical bone; Type II—defined by a thick cortical layer enclosing dense trabecular bone; Type III—consisting of a thin cortical layer surrounding dense trabecular bone; and Type IV—represented by a thin cortical layer enclosing sparse trabecular bone [90] (Figure 4). It has been reported that the primary stability of implants is largely derived from the mechanical interlocking of the implant with the cortical bone, and that increasing cortical bone thickness correlating with greater implant stability, making Type I and Type II alveolar bones the most favorable clinical conditions [91]. In severely suboptimal sparse maxillary bone, particularly in Type IV alveolar bone, surgical bone augmentation is indicated to ensure sufficient cortical bone prior to implant placement.

Osseous regeneration at implant sites with horizontal or vertical bone deficiencies can be achieved using guided bone regeneration (GBR). Successful regeneration requires the presence or recruitment of osteoblast precursors and growth factors, which may originate from the graft, the recipient bone bed, or surrounding vasculature. Adequate graft vascularization facilitates osteoblast-mediated bone deposition, while growth factors support osteoblast differentiation and function. Graft materials are classified as natural transplants—autografts, allografts, and xenografts—or synthetic alloplasts, and the choice of graft depends on the osteogenic potential determined by the quality and quantity of the bone matrix in the material [92,93].

Proceeding to the alveolar bone quantity, CBCT serves a fundamental role in the diagnostic analysis of the bone quantity three-dimensionally to give accurate measurements of the length, height, and width of the available bone.

In the anterior maxilla, due to the frequent occurrence of insufficient tissue volumes, buccal bone augmentation is proposed as an effective technique to maintain satisfactory peri-implant mucosal architecture for immediate implant placement, commonly indicated by patient demand driven by aesthetic considerations. As demonstrated by Capelli (2013) [94], the combination of internal and external grafting in sockets with a distance of less than 4 mm between the implant surface and the buccal plate can achieve a successful esthetic outcome [95]. The addition of a connective tissue graft to immediate placement further preserves the position of the buccal soft tissues. Yet, wound failure occurred at 26% of immediate implant placement sites, almost 5 times more frequently than in delayed implant placement due to multiple factors, namely difficulty in obtaining primary closure at the palatal aspect, obliteration of the periodontal ligament plexus following dental implant placement, and compromised healing of augmented bone. Hence, more focus should be on the minimization of surgical complications and optimization of wound healing outcomes to achieve optimal surgical outcomes [96].

However, in addition to anatomical challenges, aesthetic considerations constitute a critical aspect of treatment planning, as the achievement of favorable esthetic outcomes is closely linked to patient satisfaction and long-term acceptance of the prosthetic rehabilitation. The emergence profile, shade, shape, and size of the prosthesis, as well as the gingival tissue aesthetics (e.g., level of the gingival margin and gingival papilla) necessitate systematic evaluation and meticulous planning before the initiation of dental implant placement. CBCT is a non-invasive measurement method for the investigation of gingival thickness and biologic width in the aesthetic zone (maxillary central and lateral incisors), providing a basis for forecasting aesthetic outcomes via the gingival geometric ratio, calculated by dividing the gingival thickness at 1 mm (x) by the gingival thickness at 2 mm (y) measured from the free gingival margin (x/y). The closer the ratio is to 1, the more it indicates a more uniform gingival thickness, which projects a better aesthetic outcome. On the other hand, the shorter the distance from the gingival crest to the alveolar crest, the higher the susceptibility to gingival recession, elevating the risk of more facial tissue loss, and could result in a more compromised aesthetic outcome [97].

In the posterior maxilla, deficiency in alveolar bone with respect to the increased size of the maxillary sinus is the major obstacle in immediate implant placement. Estimations of the bone graft volume needed could be determined based on the augmentation site, elevation height, and sinus width from the CBCT images obtained [98]. Typically, in patients with appropriate residual bone height, augmentation of the sinus floor could be accomplished via the transalveolar approach using the osteotome technique. This minimally invasive procedure elevates the maxillary sinus floor through the alveolar crest using a concave-tipped tapered osteotome and increases implant survival up to 96.9% when the residual bone is greater than or equal to 5 mm [99]. For patients with planned implant placement in residual bone height of less than 5 mm, clinical studies of maxillary sinus augmentation have reported the importance of placing a collagen barrier membrane in the open window to prevent connective tissues from penetrating into the grafted bone material and to maintain the elevation of the grafted bone [100]. Apart from the lateral window technique using the Caldwell Luc approach followed by simultaneous implant placement that has become a reliable and predictable procedure for maxillary sinus floor augmentation with secured prognosis over the years [101], delayed implant placement may offer a slightly higher survival benefit in selected patients with other existing dental and medical comorbidities [102].

**Figure 5:**
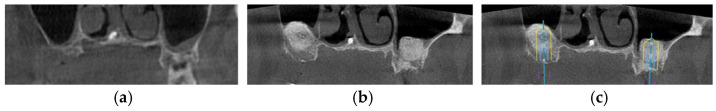
The CBCT images demonstrate an approach for implant placement in cases involving pneumatized maxillary sinuses: (**a**) the presence of enlarged bilateral maxillary sinuses necessitates bone augmentation to provide sufficient support for implant insertion; (**b**) images acquired after the bone augmentation procedure confirm successful grafting and increased bone volume; (**c**) digital implant planning software subsequently verifies that with the augmented bone, optimal implant positioning and stable placement are achievable.

**Figure 6:**
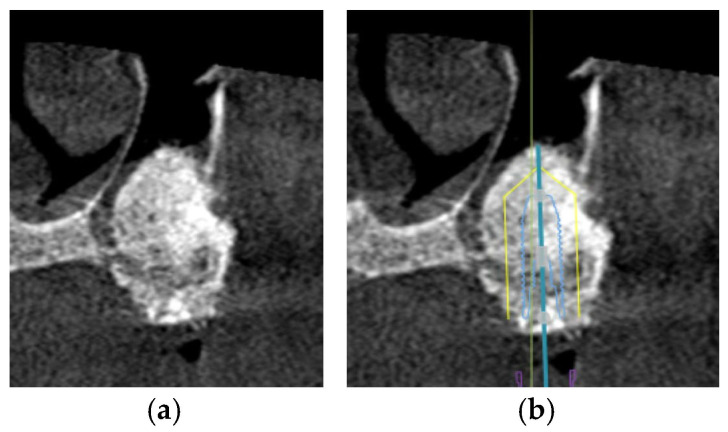
The CBCT images illustrate the treatment approach for implant placement in cases involving the pneumatized maxillary sinus: (**a**) the presence of bone graft material within the pneumatized left maxillary sinus enhances the available bone volume, facilitating successful implant placement; (**b**) digital planning software confirms that the implant can be accurately positioned within the alveolar bone, and supported by the augmented maxillary sinus.

In realistic clinical situations, recent CBCT studies found that bone graft volume decreased by an average of 25% after 4.7–6 months of healing. From 6 months after grafting until 3 years after grafting, the graft volume could be reduced by an average of 27.4–39.3% [88]. To minimize resorption of graft materials and preserve bone volume, deproteinized bovine bone mineral with a collagen membrane has been preferred over other graft materials to enhance implant stability while maintaining the contour of the maxilla [103] (Figure 5 and Figure 6).

## 6. Limitations of CBCT in Implantology

Despite the significant advantages of CBCT and its widespread integration into implant therapy, its limitations remain insufficiently addressed.

The production of artifacts can compromise diagnostic accuracy by degrading image quality. In particular, artifacts arising from beam hardening—an effect caused by the polyenergetic nature of the X-ray beam used in CBCT—may produce hypodense or hyperdense streaks and bands that obscure fine anatomical details of the alveolar bone. Complete photon absorption, or “photon starvation”, which occurs along beam paths where all photons are absorbed by dense materials, further complicates the visualization of structures adjacent to high-density objects. Together, these artifacts underscore the need for a thorough understanding of their mechanisms and for strategies to mitigate their impact, thereby ensuring accurate clinical interpretation of CBCT scans in dental and maxillofacial imaging [104].

Voxel size is another critical parameter that affects volumetric measurements. To strike a balance between diagnostic image resolution and patient safety, the selection of an appropriate voxel size is essential. An optimized voxel dimension ensures that radiation exposure is minimized while still providing sufficient image detail to support accurate implant planning. Increasing the voxel size to 0.3 mm or greater has been shown to reduce measurement accuracy and is associated with decreased inter-class reliability as well as lower intra-observer reproducibility. While CBCT hardware and voxel size alone did not meaningfully alter volumetric accuracy, the choice of segmentation software was proved to be a major determinant of accuracy, highlighting its pivotal impact on the reliability of clinical assessments [105].

In view of the significant limitations outlined, the indication for CBCT must be firmly substantiated, and any selected imaging protocol should be meticulously calibrated to ensure diagnostic value while minimizing patient radiation burden.

## 7. Future Developments of CBCT in Implantology

The widespread incorporation of artificial intelligence (AI) in radiographic interpretation has been slowly emerging into the modern field of dental implantology due to its contribution to detailed implant planning and interpretation of anatomical data. The AI-assisted detection of anatomical variations in the surrounding structures of the implant placement site can mitigate diagnostic and detection errors, further raising the success rate of dental implant surgery [106]. Not only could surgical accuracy be raised, but time spent on surgical planning could also be significantly reduced with the use of AI.

A major limitation of generic models on 2D images is their inability to convey the true morphology, volume, and spatial location of detected lesions—information essential for planning appropriate surgical interventions in the sinus region. Manual identification and cropping works are also time-consuming, further limiting the implementation of these models for routine clinical use. However, with the 3D convolutional neural network (CNN), a class of artificial neural networks that has been frequently used for image-related tasks, it shows no significant differences in the measurements between automated and manual measurements on CBCT, which has also been reported to show high performance in automatic detection, segmentation, and measurement of mucosal thickening and mucous retention cysts of the sinus. The identification of maxillary sinus lesions, evaluation of the extent of sinus opacification, and the surgical planning in the sinus region have also been made possible with the 3D CNN model, while achieving high classification accuracy of up to 90% [107].

Looking ahead, the continued evolution of CBCT is likely to be shaped by the integration of AI, which promises to further enhance diagnostic reliability and accuracy. By enabling automated image interpretation, reducing clinician-dependent variability and improving the detection of subtle anatomical features, AI-driven approaches have the potential to transform CBCT into a more precise and efficient diagnostic tool in the future.

As the technology further matures, the integration of digital diagnostic imaging and automated/computer-assisted workflows for implant placement is the core concept underlying what is currently termed “implant dentistry 4.0” (Stamenkovic et al., 2021 [108]). However, limitations in the availability and quality of digital information have significantly hindered its preoperative clinical application. With optimization of digital model generation to correspond to CBCT images with adequate spatial resolution and minimal noise and artefacts, appropriate segmentation and co-registration of diverse image volumes may become feasible in the near future [109]. As these technologies advance, their clinical adoption will raise standards of reliability, solidifying CBCT’s essential role in advanced dental and maxillofacial care.

## 8. Conclusions

Over the past decade, a continuum of research has been conducted to increase the accuracy of dental implant placement while optimizing the aesthetic result. Since successful treatment outcome with minimal surgical complications depends highly on the achieved implant stability, utilization of CBCT to project the 3D position of the implant in the alveolar bone as well as the horizontal and vertical requirements of implant placement must be taken into careful consideration. Especially in patients whose alveolar bone condition may lead to controversial treatment approaches or pose challenges for implant placement, CBCT enables accurate visualization of bone dimensions, further underscoring its importance as a diagnostic tool in implant therapy—a consideration that was previously missing in this discussion. As mentioned, clinicians should consider case selection to determine whether CBCT should be obtained in addition to PR to avoid misplacement and subsequent surgical complications.

Given the steadily increasing number of patients seeking dental implants as a preferred modality for tooth replacement, the literature accentuates the necessity of sustained and systematic efforts to advance radiographic technologies for the precise evaluation of alveolar bone conditions. Such continued innovation remains integral to optimizing diagnostic accuracy and, ultimately, improving long-term clinical outcomes.

## Data Availability

No new data were created or analyzed in this study. Data sharing is not applicable to this article.

## Abbreviations

The following abbreviations are used in this manuscript:

CBCT	Cone-Beam Computed Tomography
PSAA	Posterior Superior Alveolar Artery
IOA	Infraorbital Artery
PLNA	Posterior Lateral Nasal Artery
MSH	Maxillary Sinus Hypoplasia
AMO	Accessory Maxillary Ostium
AAA	Antral Alveolar Artery
IF	Incidental Finding
PR	Panoramic Radiography
ALARA	As Low As Reasonably Achievable
FOV	Field of View
KMW	Keratinized Mucosa Width
GBR	Guided Bone Regeneration
AI	Artificial Intelligence
CNN	Convolutional Neutral Network

## References

[1] Sharma S., Ravi Kiran S., Kumar P., Shankar R., Kumar Upadhyay A. (2024). Per-Ingvar Brånemark (1929–2014): A Homage to the Father of Osseointegration and Modern Dentistry. Cureus.

[2] Vissink A., Spijkervet F., Raghoebar G.M. (2018). The medically compromised patient: Are dental implants a feasible option?. Oral Dis..

[3] Yu B., Wang C.Y. (2022). Osteoporosis and periodontal diseases—An update on their association and mechanistic links. Periodontology 2000.

[4] Sulaiman N., Fadhul F., Chrcanovic B.R. (2023). Bisphosphonates and Dental Implants: A Systematic Review and Meta-Analysis. Materials.

[5] Bhatia A.P., Rupamalini S.N., Sathi K.V., Marella V.G., Pendyala S.K., Purohit J., Tiwari R.V. (2024). Impact of the Habit of Alcohol Consumption on the Success of the Implants: A Retrospective Study. J. Pharm. Bioallied Sci..

[6] Martinez-Amargant J., de Tapia B., Pascual A., Takamoli J., Esquinas C., Nart J., Valles C. (2023). Association between smoking and peri-implant diseases: A retrospective study. Clin. Oral Implant. Res..

[7] AlRowis R., Albelaihi F., Alquraini H., Almojel S., Alsudais A., Alaqeely R. (2025). Factors Affecting Dental Implant Failure: A Retrospective Analysis. Healthcare.

[8] Moy P.K., Aghaloo T. (2019). Risk factors in bone augmentation procedures. Periodontology 2000.

[9] Taban M., Fatemi A., Soleimani M., Sajedi S.M., Sabzevari B. (2023). Risk factors associated with implant sites prepared by orthodontic treatment: A systematic review. Eur. J. Transl. Myol..

[10] Hussain R.A., Gangwani P., Miloro M. (2022). Management of Complications in Oral and Maxillofacial Surgery.

[11] Omar S., Jaiswal H., Kumar P., Mishra S.K. (2022). Surgical Considerations and Related Complications in Oral Implantology: A Comprehensive Review. J. Prim. Care Dent. Oral Health.

[12] Bromberg N., Brizuela M. (2023). Dental Cone Beam Computed Tomography. StatPearls.

[13] Putra R.H., Cooray U., Nurrachman A.S., Yoda N., Judge R., Putri D.K., Astuti E.R. (2024). Radiographic alveolar bone assessment in correlation with primary implant stability: A systematic review and meta-analysis. Clin. Oral Implant. Res..

[14] Hussaini S., Glogauer M., Sheikh Z., Al-Waeli H. (2024). CBCT in Dental Implantology: A Key Tool for Preventing Peri-Implantitis and Enhancing Patient Outcomes. Dent. J..

[15] Elraee L., Ibrahim S.S.A., Adel-Khattab D. (2024). Double layer graft technique for horizontal alveolar ridge augmentation with staged implant placement: Radiographic histological and implant stability analysis-a case report. BMC Oral Health.

[16] Vaquette C., Mitchell J., Ivanovski S. (2022). Recent Advances in Vertical Alveolar Bone Augmentation Using Additive Manufacturing Technologies. Front. Bioeng. Biotechnol..

[17] Morgan N., Meeus J., Shujaat S., Cortellini S., Bornstein M.M., Jacobs R. (2023). CBCT for Diagnostics, Treatment Planning and Monitoring of Sinus Floor Elevation Procedures. Diagnostics.

[18] Sofia R., Abecasis P., Jaureguy S., Miravé A.M. (2024). Importance of Cbct In Implantology in the Pre—And Post-surgical Phase. Preprints.

[19] Calin F., Dalewski B., Ellmann M., Kiczmer P., Ihde S., Bieńkowska M., Kotuła J., Pałka Ł. (2025). CBCT Evaluation of Maxillary Incisive Canal Characteristics Among Population in Regard to Possibility of Implant Cortical Anchorage-A Multicenter Study. Dent. J..

[20] Gönül Y., Bucak A., Atalay Y., Beker-Acay M., Çalişkan A., Sakarya G., Soysal N., Cimbar M., Özbek M. (2016). MDCT evaluation of nasopalatine canal morphometry and variations: An analysis of 100 patients. Diagn. Interv. Imaging.

[21] Fernandes J., Rohinikumar S., Nessapan T., Rani D., Abhinav R.P., Gajendran P. (2022). CBCT Analysis of Prevalence of the Canalis Sinuosus on the Alveolar Ridge in the Site of Endosseous Implant Placement: A Retrospective Study. J. Long-Term Eff. Med. Implant..

[22] Lopes Dos Santos G., Ikuta C.R.S., Salzedas L.M.P., Miyahara G.I., Tjioe K.C. (2020). Canalis sinuosus: An Anatomic Repair that May Prevent Success of Dental Implants in Anterior Maxilla. J. Prosthodont. Off. J. Am. Coll. Prosthodont..

[23] Shan T., Qu Y., Huang X., Gu L. (2021). Cone beam computed tomography analysis of accessory canals of the canalis sinuosus: A prevalent but often overlooked anatomical variation in the anterior maxilla. J. Prosthet. Dent..

[24] Mularczyk C., Welch K. (2024). Maxillary Sinus Anatomy and Physiology. Otolaryngol. Clin. N. Am..

[25] Henson B., Drake T.M., Edens M.A. (2023). Anatomy, Head and Neck, Nose Sinuses. StatPearls.

[26] Yılmaz İ., Lafci Fahrioglu S., Firincioglulari M., Orhan K., İlgi S. (2025). Examination and Relationship of Posterior Superior Alveolar Artery and Canalis Sinuosus Using Cone Beam CT. Biomimetics.

[27] Gulec M., Icen V., Ozmen E.E. (2025). Evaluation of maxillary sinus pathologies and the posterior superior alveolar artery canal using cone-beam computed tomography. BMC Oral Health.

[28] Çam K., Zengin A.Z. (2025). Evaluation of the location of posterior superior alveolar artery and infraorbital foramen originating from the same source by using cone beam computed tomography. BMC Oral Health.

[29] Nguyen J.D., Duong H. (2022). Anatomy, Head and Neck, Lateral Nasal Artery. StatPearls.

[30] Shafique S., Das J.M. (2023). Anatomy, Head and Neck, Maxillary Nerve. StatPearls.

[31] Patel R., Clarkson E. (2021). Implant Surgery Update for the General Practitioner: Dealing with Common Postimplant Surgery Complications. Dent. Clin. N. Am..

[32] Papadopoulou A.M., Chrysikos D., Samolis A., Tsakotos G., Troupis T. (2021). Anatomical Variations of the Nasal Cavities and Paranasal Sinuses: A Systematic Review. Cureus.

[33] Dogan M.E., Uluısık N., Yuvarlakbaş S.D. (2024). Retrospective analysis of pathological changes in the maxillary sinus with CBCT. Sci. Rep..

[34] Lim H.C., Kim S., Kim D.H., Herr Y., Chung J.H., Shin S.I. (2021). Factors affecting maxillary sinus pneumatization following posterior maxillary tooth extraction. J. Periodontal Implant. Sci..

[35] Yücesoy T., Göktaş T.A. (2022). Evaluation of Sinus Pneumatization and Dental Implant Placement in Atrophic Maxillary Premolar and Molar Regions. Int. J. Oral Maxillofac. Implant..

[36] Amine K., Slaoui S., Kanice F.Z., Kissa J. (2020). Evaluation of maxillary sinus anatomical variations and lesions: A retrospective analysis using cone beam computed tomography. J. Stomatol. Oral Maxillofac. Surg..

[37] Souza D.A.S., Costa F.W.G., de Mendonça D.S., Ribeiro E.C., de Barros Silva P.G., Neves F.S. (2024). Computed tomography assessment of maxillary sinus hypoplasia and associated anatomical variations: A systematic review and meta-analysis of global evidence. Oral Radiol..

[38] Atsal G., Demir E., Yildirim O., Edizer D.T., Olgun L. (2022). The Relationship Between Degree of Nasal Septum Deviation with Sinonasal Structures and Variations. J. Craniofacial Surg..

[39] Park W.B., Sadilina S., Han J.Y., Thoma D.S., Lim H.C. (2025). Maxillary sinus hypoplasia relevant to dental implant treatment: A narrative review. J. Periodontal Implant. Sci..

[40] Do J., Han J.J. (2022). Anatomical Characteristics of the Accessory Maxillary Ostium in Three-Dimensional Analysis. Medicina.

[41] Nguyen P.N., Kruger E., Huang T., Koong B. (2020). Incidental findings detected on cone beam computed tomography in an older population for pre-implant assessment. Aust. Dent. J..

[42] Iușan S.A.L., Costache C., Lucaciu O.P., Petrescu B.N., Mirică I.C., Toc D.A., Albu S. (2023). Correlations between Dental Implant Infectious Pathologies and Maxillary Sinusitis: A Review Article. J. Clin. Med..

[43] Safi Y., Amid R., Zadbin F., Ghazizadeh Ahsaie M., Mortazavi H. (2021). The occurrence of dental implant malpositioning and related factors: A cross-sectional cone-beam computed tomography survey. Imaging Sci. Dent..

[44] Hui L., Hung K.F., Yeung A.W.K., von Arx T., Leung Y.Y., Bornstein M.M. (2022). Anatomical variations of the ethmoid sinuses and their association with health or pathology of the ethmoid and maxillary sinuses in a Southern Chinese population: An analysis using cone-beam computed tomography. Imaging Sci. Dent..

[45] Staněk J., Machálková K., Staňková M., Zapletalová J., Kocurová T. (2023). Alveolar antral artery: Cone beam computed tomography study and clinical context. PeerJ.

[46] Alshamrani A.M., Mubarki M., Alsager A.S., Alsharif H.K., AlHumaidan S.A., Al-Omar A. (2023). Maxillary Sinus Lift Procedures: An Overview of Current Techniques, Presurgical Evaluation, and Complications. Cureus.

[47] Lakshmi Y.V., Singh T.D., Fathima R., Kumar V.H. (2021). Incidence of concha bullosa and its role in chronic rhinosinusitis. Int. J. Otorhinolaryngol. Head Neck Surg..

[48] Othman B., Zahid T. (2022). Mental Nerve Anterior Loop Detection in Panoramic and Cone Beam Computed Tomography Radiograph for Safe Dental Implant Placement. Cureus.

[49] Bertram A., Eckert A.W., Emshoff R. (2021). Implant-to-root dimensions projected by panoramic radiographs in the maxillary canine-premolar region: Implications for dental implant treatment. BMC Med. Imaging.

[50] Araujo G.T.T., Peralta-Mamani M., Silva A.F.M.D., Rubira C.M.F., Honório H.M., Rubira-Bullen I.R.F. (2019). Influence of cone beam computed tomography versus panoramic radiography on the surgical technique of third molar removal: A systematic review. Int. J. Oral Maxillofac. Surg..

[51] Fischborn A.R., Andreis J.D., Wambier L.M., Pedroso C.M., Claudino M., Franco G.C.N. (2023). Performance of panoramic radiography compared with computed tomography in the evaluation of pathological changes in the maxillary sinuses: A systematic review and meta-analysis. Dentomaxillofac. Facial Radiol..

[52] Watanabe H., Ariji Y., Fukuda M., Kuwada C., Kise Y., Nozawa M., Sugita Y., Ariji E. (2020). Deep learning object detection of maxillary cyst-like lesions on panoramic radiographs: Preliminary study. Oral. Radiol..

[53] Hung K.F., Ai Q.Y.H., King A.D., Bornstein M.M., Wong L.M., Leung Y.Y. (2022). Automatic detection and segmentation of morphological changes of the maxillary sinus mucosa on cone-beam computed tomography images using a three-dimensional convolutional neural network. Clin. Oral Investig..

[54] Lee J.H., Yun J.H., Kim Y.T. (2024). Deep learning to assess bone quality from panoramic radiographs: The feasibility of clinical application through comparison with an implant surgeon and cone-beam computed tomography. J. Periodontal Implant. Sci..

[55] Vyas R., Khurana S., Khurana D., Singer S.R., Creanga A.G. (2023). Cone Beam Computed Tomography (CBCT) Evaluation of Alveolar Bone Thickness and Root Angulation in Anterior Maxilla for Planning Immediate Implant Placement. Cureus.

[56] Bertram A., Eckert A.W., Emshoff R. (2022). Implant-to-nasal floor dimensions projected by panoramic radiographs in the maxillary incisor-canine region: Implications for dental implant treatment. Odontology.

[57] Tadinada A., Proft B., Thacker S., Yadav S. (2023). Comparative Evaluation of a Lower-Dose CBCT Acquisition Protocol for Preoperative Implant Site Assessment in Dry Human Skulls: A Proof-of-Concept Study. J. Oral Implantol..

[58] Hamilton A., Singh A., Friedland B., Jamjoom F.Z., Griseto N., Gallucci G.O. (2022). The impact of cone beam computer tomography field of view on the precision of digital intra-oral scan registration for static computer-assisted implant surgery: A CBCT analysis. Clin. Oral Implant. Res..

[59] Kehrwald R., Castro H.S., Salmeron S., Matheus R.A., Santaella G.M., Queiroz P.M. (2022). Influence of Voxel Size on CBCT Images for Dental Implants Planning. Eur. J. Dent..

[60] Sawicki P., Regulski P., Winiarski A., Zawadzki P.J. (2022). Influence of Exposure Parameters and Implant Position in Peri-Implant Bone Assessment in CBCT Images: An In Vitro Study. J. Clin. Med..

[61] Motel C., Kirschner C., Förtsch F., Buchbender M., Wichmann M., Matta R.E. (2025). The influence of the superimposition procedure and type of intraoral impression on the superimposition accuracy of CBCT scans with dental impressions in implant planning: An in-vitro study. Int. J. Implant. Dent..

[62] Ntovas P., Marchand L., Finkelman M., Revilla-León M., Att W. (2024). Accuracy of manual and artificial intelligence-based superimposition of cone-beam computed tomography with digital scan data, utilizing an implant planning software: A randomized clinical study. Clin. Oral Implant. Res..

[63] Mizuno K., Nakano T., Shimomoto T., Fujita Y., Ishigaki S. (2022). The efficacy of immediate implant placement in the anterior maxilla with dehiscence in the facial alveolar bone: A case series. Clin. Implant. Dent. Relat. Res..

[64] Gupta G., Gupta D.K., Gupta N., Gupta P., Rana K.S. (2019). Immediate Placement, Immediate Loading of Single Implant in Fresh Extraction Socket. Contemp. Clin. Dent..

[65] Chatzopoulos G.S., Wolff L.F. (2022). Survival Rates and Factors Affecting the Outcome Following Immediate and Delayed Implant Placement: A Retrospective Study. J. Clin. Med..

[66] Strauss F.J., Gil A., Smirani R., Rodriguez A., Jung R., Thoma D. (2024). The use of digital technologies in peri-implant soft tissue augmentation—A narrative review on planning, measurements, monitoring and aesthetics. Clin. Oral Implant. Res..

[67] Ravidà A., Arena C., Tattan M., Caponio V.C.A., Saleh M.H.A., Wang H.L., Troiano G. (2022). The role of keratinized mucosa width as a risk factor for peri-implant disease: A systematic review, meta-analysis, and trial sequential analysis. Clin. Implant. Dent. Relat. Res..

[68] Elkhaweldi A., Rincon Soler C., Cayarga R., Suzuki T., Kaufman Z. (2015). Various Techniques to Increase Keratinized Tissue for Implant Supported Overdentures: Retrospective Case Series. Int. J. Dent..

[69] Resnik D.M.D.S. (2020). Misch’s contemporary implant dentistry e-book. Misch’s Contemporary Implant Dentistry e-Book.

[70] Janjua O.S., Qureshi S.M., Shaikh M.S., Alnazzawi A., Rodriguez-Lozano F.J., Pecci-Lloret M.P., Zafar M.S. (2022). Autogenous Tooth Bone Grafts for Repair and Regeneration of Maxillofacial Defects: A Narrative Review. Int. J. Environ. Res. Public Health.

[71] Heimes D., Schiegnitz E., Kuchen R., Kämmerer P.W., Al-Nawas B. (2021). Buccal Bone Thickness in Anterior and Posterior Teeth-A Systematic Review. Healthcare.

[72] Cruz R.S., Lemos C.A.A., de Luna Gomes J.M., Fernandes EOliveira H.F., Pellizzer E.P., Verri F.R. (2022). Clinical comparison between crestal and subcrestal dental implants: A systematic review and meta-analysis. J. Prosthet. Dent..

[73] Comuzzi L., Ceddia M., Di Pietro N., Inchingolo F., Inchingolo A.M., Romasco T., Tumedei M., Specchiulli A., Piattelli A., Trentadue B. (2023). Crestal and Subcrestal Placement of Morse Cone Implant-Abutment Connection Implants: An In Vitro Finite Element Analysis (FEA) Study. Biomedicines.

[74] Pradhan Y., Srivastava G., Choudhury G.K., Sahoo P.K., Padhiary S.K. (2024). Short implant versus conventional implant in the posterior atrophic maxilla: A systematic review and meta-analysis. J. Indian Prosthodont. Soc..

[75] Mistry A., Ucer C., Thompson J.D., Khan R.S., Karahmet E., Sher F. (2021). 3D Guided Dental Implant Placement: Impact on Surgical Accuracy and Collateral Damage to the Inferior Alveolar Nerve. Dent. J..

[76] Renton T. (2010). Prevention of iatrogenic inferior alveolar nerve injuries in relation to dental procedures. Dental update.

[77] Raj I., Harinee A., Raj A.S., Uikey A.K., Syed F. (2024). Enhancing Anterior Esthetic Zone Implant Placement Through Bone Manipulation Techniques: A Case Series. Cureus.

[78] Goldstein G., Goodacre C., Brown M.C., Tarnow D.P. (2024). Proposal regarding potential causes related to certain complications with dental implants and adjacent natural teeth: Physics applied to prosthodontics. J. Prosthodont. Off. J. Am. Coll. Prosthodont..

[79] Ayman D., Shawky M., Aly L.A.A., Mounir M., Zekry A.K.A. (2025). Bone gain and accuracy assessment of computer-guided workflow for horizontal augmentation of atrophic anterior maxilla with symphyseal cortical plates: A randomized controlled trial. BMC Oral Health.

[80] Andre A., Ogle O.E. (2021). Vertical and Horizontal Augmentation of Deficient Maxilla and Mandible for Implant Placement. Dent. Clin. N. Am..

[81] Flanagan D. (2021). Rationale for Mini Dental Implant Treatment. J. Oral Implantol..

[82] Ceddia M., Marchioli G., Romasco T., Comuzzi L., Piattelli A., Deporter D.A., Di Pietro N., Trentadue B. (2025). Effect of Crestal Position on Bone-Implant Stress Interface of Three-Implant Splinted Prostheses: A Finite Element Analysis. Materials.

[83] Mozaffari A., Hashtbaran D., Moghadam A., Aalaei S. (2023). Stress Distribution in Peri-implant Bone in the Replacement of Molars with One or Two Implants: A Finite Element Analysis. J. Dent..

[84] Jia-Mahasap W., Rungsiyakull C., Bumrungsiri W., Sirisereephap N., Rungsiyakull P. (2022). Effect of Number and Location on Stress Distribution of Mini Dental Implant-Assisted Mandibular Kennedy Class I Removable Partial Denture: Three-Dimensional Finite Element Analysis. Int. J. Dent..

[85] Flanagan D. (2024). Horizontal Alveolar Ridge Splitting and Expansion. J. Oral Implantol..

[86] Kim D., Kim K., Ohe J.Y., Song S.J., Paek J. (2025). Correlation between implant angulation and crestal bone changes: A 5-year retrospective study. J. Prosthet. Dent..

[87] Bokobza A., Lauwers L., Raoul G., Nicot R., Ferri J. (2022). Implant repositioning with segmental osteotomy. J. Stomatol. Oral Maxillofac. Surg..

[88] Yeung A.W.K., Hung K.F., Li D.T.S., Leung Y.Y. (2022). The Use of CBCT in Evaluating the Health and Pathology of the Maxillary Sinus. Diagnostics.

[89] Rosas-Díaz J.C., Córdova-Limaylla N.E., Palomino-Zorrilla J.J., Guerrero M.E., Carreteros R., Cervantes-Ganoza L.A., Cayo-Rojas C.F. (2022). Repeatability and Reproducibility of a Modified Lekholm and Zarb Bone Quality Classification Based on Cone Beam Computed Tomography: An Observatsion Study. J. Int. Soc. Prev. Community Dent..

[90] Juodzbalys G., Kubilius M. (2013). Clinical and radiological classification of the jawbone anatomy in endosseous dental implant treatment. J. Oral Maxillofac. Res..

[91] Kim M., Lee J.H., Kim H.S., Lee S.Y. (2025). Evaluation of primary stability of implants in bovine bone defects models. Sci. Rep..

[92] Sheikh Z., Sima C., Glogauer M. (2015). Bone Replacement Materials and Techniques Used for Achieving Vertical Alveolar Bone Augmentation. Materials.

[93] Liu J., Kerns D.G. (2014). Mechanisms of guided bone regeneration: A review. Open Dent. J..

[94] Capelli M., Testori T., Galli F., Zuffetti F., Motroni A., Weinstein R., Del Fabbro M. (2013). Implant-buccal plate distance as diagnostic parameter: A prospective cohort study on implant placement in fresh extraction sockets. J. Periodontol..

[95] Chen K., Li Z., Liu X., Liu Q., Chen Z., Sun Y., Chen Z., Huang B. (2021). Immediate Implant Placement with Buccal Bone Augmentation in the Anterior Maxilla with Thin Buccal Plate: A One-Year Follow-Up Case Series. J. Prosthodont. Off. J. Am. Coll. Prosthodont..

[96] Wu X.Y., Shi J.Y., Buti J., Lai H.C., Tonetti M.S. (2023). Buccal bone thickness and mid-facial soft tissue recession after various surgical approaches for immediate implant placement: A systematic review and network meta-analysis of controlled trials. J. Clin. Periodontol..

[97] Chow R.L.K., Lau S.L., Leung Y.Y., Chow J.K.F. (2023). A non-invasive method for the assessment of gingival thickness in the aesthetic zone and the concept of the gingival geometric ratio in an Asian population. Int. J. Oral Maxillofac. Surg..

[98] Hung K.F., Hui L.L., Leung Y.Y. (2022). Patient-specific estimation of the bone graft volume needed for maxillary sinus floor elevation: A radiographic study using cone-beam computed tomography. Clin. Oral Investig..

[99] Molina A., Sanz-Sánchez I., Sanz-Martín I., Ortiz-Vigón A., Sanz M. (2022). Complications in sinus lifting procedures: Classification and management. Periodontology 2000.

[100] Yamaguchi K., Munakata M., Sato D., Kataoka Y., Kawamata R. (2023). The Effectiveness and Practicality of a Novel Barrier Membrane for the Open Window in Maxillary Sinus Augmentation with a Lateral Approach, with Risk Indicators for Bone Graft Displacement and Bone Height Decrease: A Prospective Study in Humans. Bioengineering.

[101] Reis I.N.R.D., Jue A., Wolvius E., Pijpe J., Spin-Neto R., Jung R.E., Naenni N., Strauss F.J., Jonker B. (2025). Analysis of bone dimensional stability after two-stage maxillary sinus floor augmentation with autogenous bone versus bovine bone mineral combined with autogenous bone chips: Results from a 1-year multicenter split-mouth randomized controlled trial. J. Dent..

[102] Patel R., Ucer C., Wright S., Khan R.S. (2023). Differences in Dental Implant Survival between Immediate vs. Delayed Placement: A Systematic Review and Meta-Analysis. Dent. J..

[103] Mahardawi B., Jiaranuchart S., Arunjaroensuk S., Dhanesuan K., Mattheos N., Pimkhaokham A. (2025). The clinical benefit of alveolar ridge preservation in the posterior maxilla: A systematic review and meta-analysis. Sci. Rep..

[104] da Fonte J.B.M., Fontenele R.C., Freitas D.Q. (2024). Expression of beam hardening artifacts on horizontally stitched cone-beam computed tomography images. Imaging Sci. Dent..

[105] Ozdede M., Akay G., Karadag Atas O. (2025). Influence of CBCT device, voxel size, and segmentation software on the accuracy of tooth volume measurements. BMC Oral Health.

[106] Kurt Bayrakdar S., Orhan K., Bayrakdar I.S., Bilgir E., Ezhov M., Gusarev M., Shumilov E. (2021). A deep learning approach for dental implant planning in cone-beam computed tomography images. BMC Med. Imaging.

[107] Hung K.F., Ai Q.Y.H., Wong L.M., Yeung A.W.K., Li D.T.S., Leung Y.Y. (2022). Current Applications of Deep Learning and Radiomics on CT and CBCT for Maxillofacial Diseases. Diagnostics.

[108] Stamenkovic D., Obradovic-Duricic K., Stamenkovic D., Grbovic A., Dordevic I. (2021). The fourth industrial Revolution's impact on dentistry. Srpski Arhiv za Celokupno Lekarstvo.

[109] Fuglsig J.M.C.E.S., Reis I.N.R.D., Yeung A.W.K., Bornstein M.M., Spin-Neto R. (2024). The current role and future potential of digital diagnostic imaging in implant dentistry: A scoping review. Clin. Oral Implant. Res..

